# Effect of Warm Climate on Pulmonary Diseases

**Published:** 1845-11

**Authors:** 


*Effect of Warm Climate on Pulmonary Disease.*—Baron Larrey disagrees with most of our medical men in his opinion of the effects of climate on pulmonary disease. He says:

It is a very fatal error, or perhaps rather a pernicious prejudice, for medical men to imagine that mild and warm climates can effect a cure of thoracic diseases. The cold and humidity, which generally are the determining causes of these affections, doubtless aggravate their symptoms; but when the mischief has reached the second stage of its career, atmospheric heat almost always does harm instead of good, and very generally accelerates the fatal issue. It is the same with phthisis as with all chronic maladies that are attended with hypertrophy or engorgement of the parenchymatous or membranous viscera.—*Medico-Chirurgical Review.—Braithwait's Retrospect.*

